# FOXO1 inhibits osteosarcoma oncogenesis via Wnt/β-catenin pathway suppression

**DOI:** 10.1038/oncsis.2015.25

**Published:** 2015-09-07

**Authors:** H Guan, P Tan, L Xie, B Mi, Z Fang, J Li, J Yue, H Liao, F Li

**Affiliations:** 1 Department of Orthopaedic Surgery, Tongji Hospital, Tongji Medical College, Huazhong University of Science and Technology, Wuhan, China; 2 Cancer Center of Union Hospital, Tongji Medical College, Huazhong University of Science and Technology, Wuhan, China; 3 Department of Pathology, Hubei Cancer Hospital, Wuhan, China

## Abstract

Recent advances have highlighted profound roles of FOXO transcription factors, especially FOXO1, in bone development and remodeling. The regulation of bone development by FOXOs seems to be stage-specific or context dependent. FOXOs promote maintenance and differentiation of early progenitors of the osteoblast lineage and repress proliferation of committed osteoblast precursors; FOXO1 is vital for osteocyte survival. Considering the versatile roles played by FOXOs in bone development and tumorigenesis, it is plausible that FOXO1, the main FOXO in bone with a non-redundant role, might have influence on osteosarcoma (OS) oncogenesis. Indeed, recent results have implicated that FOXO1 has a tumor-suppressing role in OS. In the present study, we found that *FOXO1* expression was generally low or absent in OS, with a minority of cases having moderate expression. Whole-genome sequencing (WGS) revealed that the *FOXO1* locus was frequently involved in copy number variation and loss of heterozygosity in OS, indicating that chromosomal aberrations might be partially responsible for the heterogeneity in *FOXO1* expression. FOXO1 activation in OS cell lines inhibited cancer cell survival, which can be attributed to modulation of target genes, including *BIM* and repressed Wnt/β-catenin signaling. FOXO1 inhibition promoted cell proliferation, enhanced colony formation and attenuated osteogenic differentiation of OS cell lines. To conclude, our results proved FOXO1 as a tumor suppressor in OS at least partially by suppression of the Wnt/β-catenin pathway.

## Introduction

Osteosarcoma (OS) is the most common primary bone cancer.^1^ The incidence of OS is 4.8 per million per year.^2^ OS is characterized by the formation of immature bone or osteoid tissue by malignant osteoblasts. A hallmark of OS pathogenesis is the high frequency of genomic reorganizations resulting in extremely complex karyotypes with very few consistent genetic findings.^2^ TP53 and RB1 signaling represents the most affected tumor-suppressing pathway.^1^ Major oncogenic pathways in OS include the canonical Wnt/β-catenin signaling, activated in OS by various mechanisms.^3^ Most OS tumors show overexpression and/or increased nuclear localization of β-catenin, which correlates with lung metastasis.^4, 5^ The *MYC* oncogene is amplified in about 10% of OS tumors, upregulated in 23–42% of OS tumors and associated with poor prognosis.^1^

Another main characteristic of OS oncogenesis is enhanced growth factors and signaling pathways. Insulin-like growth factor receptor signaling pathway is constitutively activated in human OS compared with normal osteoblasts and mesenchymal stem cells.^6^ Vascular endothelial growth factor pathway and multiple genes involved in the pathway are over-represented in OS. Moreover, vascular endothelial growth factor gene amplification and vascular endothelial growth factor expression have been shown to be associated with poor prognosis^7, 8^ and pulmonary metastasis.^8^ Platelet-derived growth factor and its receptor are expressed in most OS tumors and correlate with inferior event-free survival.^9^ These growth factors are potent mitogens for tumor cells and act by autocrine or paracrine mechanisms through modulation of multiple oncogenic signaling pathways, including suppression of FOXO transcription factors via the PI3K/AKT pathway.^10^

AKT, the effector protein of PI3K signaling and a central regulator of growth-promoting signals, phosphorylates FOXO proteins and leads to their inactivation and nucleus exclusion. FOXOs play essential roles in regulation of development and tumorigenesis. Especially, FOXOs affect longevity in various model organisms^11^ and are associated with human longevity across different populations.^12, 13^ FOXOs exert their influence on cellular processes mainly by regulating transcription of target genes, for example, apoptosis (BIM, NOXA, TRAIL), cell cycle arrest (CDKN1B, CCND1) and redox balance (SOD2 and catalase). FOXOs were also shown to reduce production of reactive oxygen species by inhibition of mitochondrial function through decreased MYC activity.^14, 15, 16^

Recent advances using mouse models highlighted profound influences of FOXOs, especially FOXO1, on bone development and remodeling.^11, 17, 18, 19, 20^ The regulation of bone development by FOXOs seems to be stage-specific or context dependent. In the early progenitors of the osteoblast lineage, FOXOs promote maintenance and differentiation by activation of Runx2 and probably inhibition of ROS.^20^ FOXOs repress proliferation of committed osteoblast precursors by antagonizing the canonical Wnt/β-catenin signaling,^11^ whereas FOXO1 is the only FOXO protein that is vital for survival of osteoblasts and osteocytes and controls bone mass through reduction of ROS generation.^17^ FOXO1 acts as a key regulator of the endocrine function of the skeleton by regulate glucose homeostasis through regulation of osteocalcin in osteoblasts, a marker for the bone formation.^21^

Considering the versatile roles played by FOXOs in tumorigenesis and bone development, it is plausible that FOXO1, the main FOXO in bone with a non-redundant role in osteoblast differentiation,^17, 18, 19, 22^ might have influence on OS pathogenesis. Indeed, recent results implicated that FOXO1 had a tumor-suppressing role in OS.^23, 24, 25^ Especially, FOXO1 was shown to be targeted by MiR-135b and expressed at low levels in OS tissues comparing to paired adjacent non-neoplastic bone; forced expression of FOXO1 inhibited OS cell proliferation and invasion.^25^

In the present study, we demonstrated that (a) *FOXO1* expression was absent or low in OS tumors; (b) activation of a conditional *FOXO1-ER* construct induced OS cell apoptosis and cell cycle arrest, and inhibited colony formation of OS cells; (c) the mechanisms could be attributed to modulation of target gene expression and attenuation of Wnt/β-catenin signaling by FOXO1; (d) knockdown of *FOXO1* expression by siRNA or use of a specific inhibitor of FOXO1 transcriptional activity promoted cell growth, enhanced colony formation and impaired osteoblast differentiation of OS cells.

## Results

### FOXO1 expression in OS

**Figure 1:**
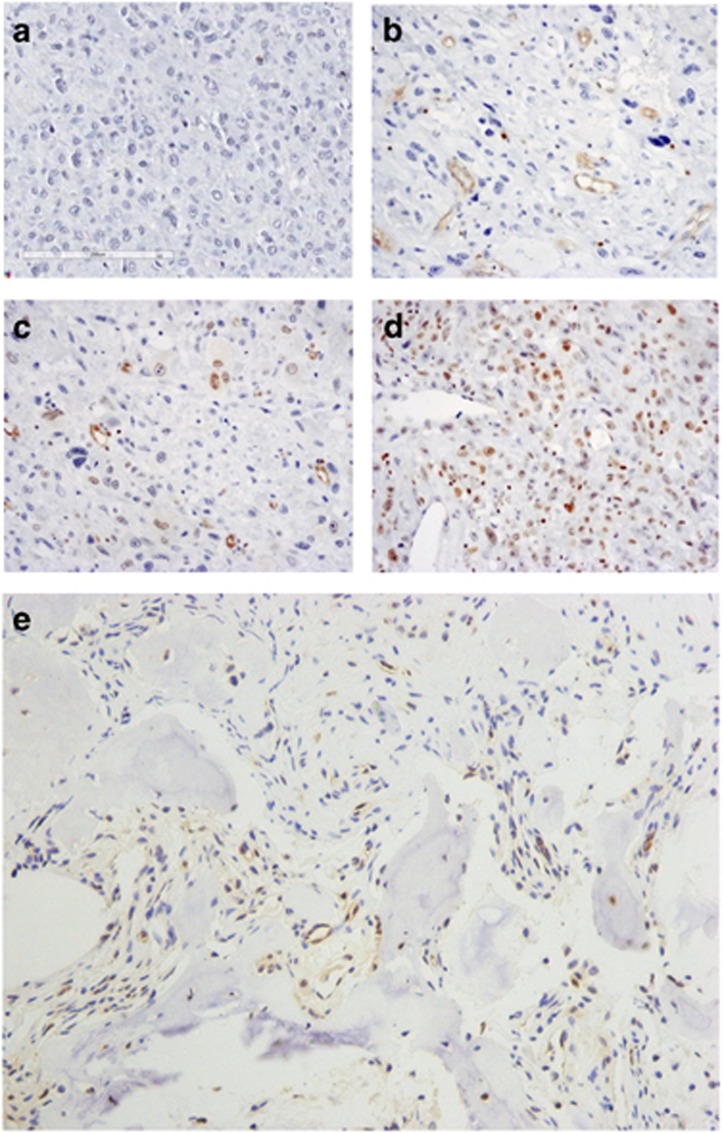
*FOXO1* expression in OS and osteoid osteoma. Original magnification × 200. (**a**–**d**) FOXO1 expression in OS. (**a**) OS cells are FOXO1-negative. (**b**) OS cells are FOXO1-negative. Vascular endothelial cells are FOXO1-positive (+). (**c**) OS cells and vascular endothelial cells are FOXO1-positive (+). (**d**) OS cells are FOXO1-positive (++). (**e**) Osteoid osteoma stained positive for FOXO1 (+).

**Figure 2:**
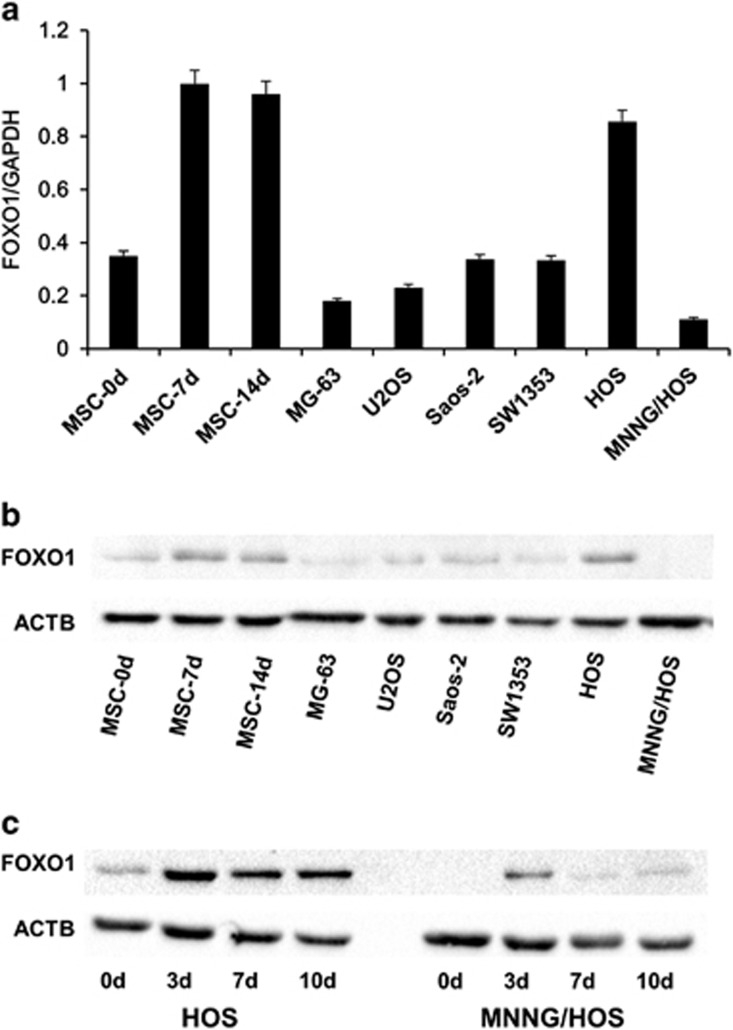
*FOXO1* expression in hMSC and OS cell lines. hMSC, HOS and MNNG/HOS cells were treated with osteogenic differentiation medium to induce osteogenic differentiation. At different time points as indicated (d stands for day), cells were collected and mRNA and protein were prepared. (**a**) Expression of *FOXO1* mRNA in hMSC and osteosarcoma cell lines was assessed by quantitative RT-PCR as described in Materials and Methods. Data are mean±s.d. of the target gene to reference gene (FOXO1/GAPDH) ratio. (**b**) Expression of *FOXO1* protein in hMSC and osteosarcoma cell lines was assessed by immunoblot. ACTB served as a loading control. (**c**) Expression of FOXO1 protein in HOS and MNNG/HOS cells treated by osteogenic differentiation medium was evaluated. ACTB was used as a loading control. The experiments were done in triplicate.

To elucidate the role of FOXO1 in OS tumorigenesis, we first evaluated its expression levels in OS compared with other sarcomas. With the help of a Oncomine software (Life Technologies, Thermo Fisher Scientific, Waltham, MA, USA), we analyzed the published gene expression data of 181 tumors including 16 types of human bone and soft tissue sarcomas.^26^ We found that expression of FOXO1 was very low in gastrointestinal stromal tumor and OS compared with other sarcomas (Supplementary Figures 1 and 3: types of sarcomas with only one specimen were not analyzed). Next, we measured FOXO1 expression in OS through immunohistochemical analysis, with osteoid osteoma (a benign bone tumor) used as a control. FOXO1 staining was detected in 13/18 (72.2%) osteoid osteoma cases; moderate or strong staining was detected in 3/18 (16.7%) cases. Whereas in 39 of 62 OS cases analyzed FOXO1 was stained negative (62.9%), only 8 cases were stained moderate positive for FOXO1 (12.9%) (Figure 1 and Table 1). Expression of FOXO1 in chondromatous, fibromatous and osteogenous areas of OS was also analyzed. In the typical areas, FOXO1 was detected in 6/14 (42.9%) chondromatous areas, 10/22 (45.5%) fibromatous areas and rarely in osteogenous areas (1/9, 11.1%) (Table 2 and Supplementary Figure 2). We then analyzed *FOXO1* expression in five human OS cell lines and one chondrosarcoma cell line. As committed osteoblast progenitors are proposed to the cells of origin in OS, we used the human marrow stromal cell (hMSC) going through osteoblastogenesis *in vitro* as a control. The expression levels of *FOXO1* in OS cell lines and hMSC was low, with the exception of HOS, which showed moderate *FOXO1* expression (Figures 2a and b). Upon induction of osteoblast differentiation, *FOXO1* expression was significantly upregulated, much higher than most OS cell lines (Figures 2b and c). Thus, *FOXO1* expression is generally negative or low in OS, with a minority of cases expressing FOXO1 at high levels.

### *FOXO1* locus is frequently involved in copy number variation and loss of heterozygosity in OS

To better understand the potential mechanisms of heterogeneity in *FOXO1* expression, we analyzed epigenetic and genetic changes of the *FOXO1* locus. We first investigated the methylation status of the *FOXO1* promoter in OS cell lines. No significant methylation was observed (data not shown). We also analyzed the five OS cell lines for mutations in the *FOXO1* coding sequence. No point mutation was found.

The human *FOXO1* gene is located in 13q14, a locus with recurrent structural and numerical aberrations in OS.^27, 28, 29^ Therefore, the whole-genome sequencing data from a recent study^30^ were analyzed. Of the 34 OS cases, the FOXO1 locus has 5 (5/34, 14.7%) gains and 6 losses (6/34, 17.6%), all of which are broad-scale copy number variations (17–218, median 83). Fifteen samples (15/34, 44.1%) showed broad loss of heterozygosity (over 10  Mb) at this locus. No point mutations or structural variations (SVs) were identified. Thus, chromosomal alterations of the FOXO1 locus is a recurrent finding in OS that might contribute to decreased gene expression in some OS cases.

### Inhibition of cell proliferation by FOXO1

**Figure 3:**
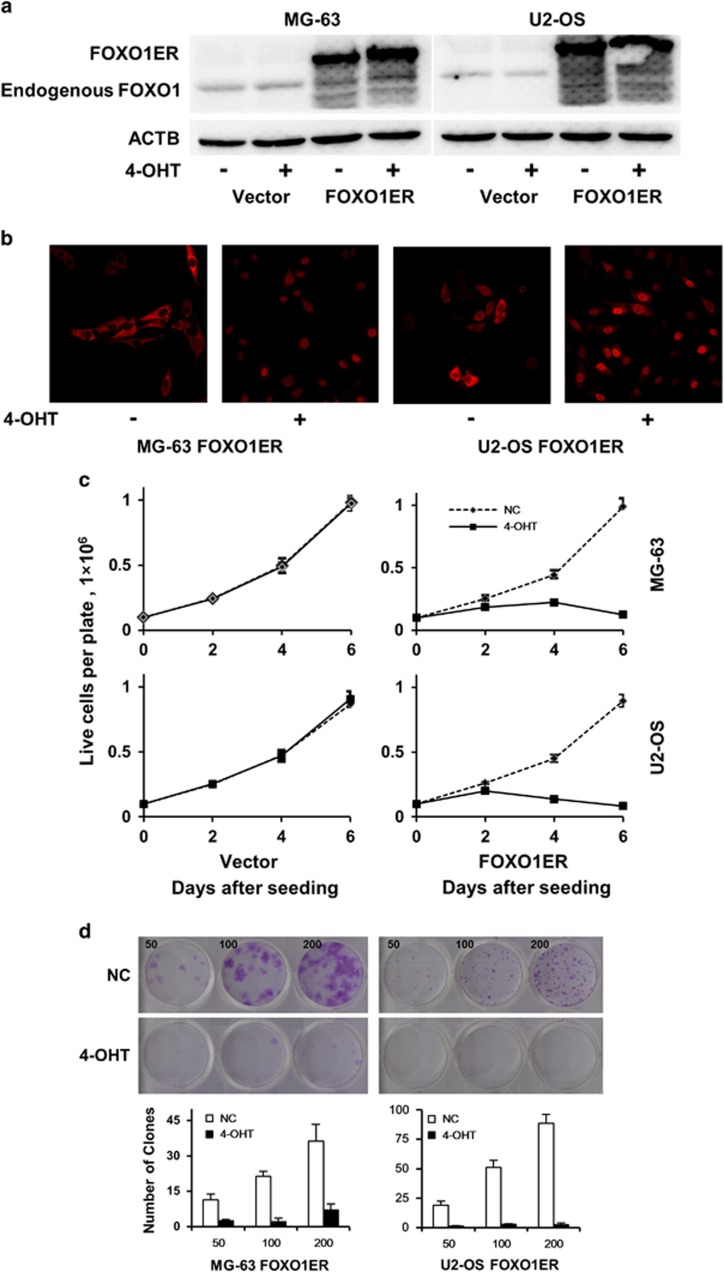
FOXO1 inhibits growth of osteosarcoma cell lines and suppresses colony formation capacity. (**a**) Expression of FOXO1ER protein in stably infected MG-63 and U2OS cells was validated by immunoblotting using anti-FOXO1 antibody. Anti-ACTB antibody was used as a loading control. (**b**) Induction of nuclear translocation of FOXO1ER by 4-OHT was verified by immunofluorescence analysis. (**c**) Cell lines were plated in six-well plates at a density of 1 × 10^5^ cells per well (day 0). 4-OHT was added every two days at a concentration of 100 nM. Cells were counted with a hemocytometer at days 2, 4 and 6. Cell viability was verified by trypan blue staining. (**d**) Representative photographs of colony formation of MG-63-FOXO1ER and U2OS-FOXO1ER cells 12 days after plating; the number of cells plated in each well is also indicated. All experiments were repeated at least three times. Colony formation was quantified and presented as mean±s.d.

The upregulation of FOXO1 during osteoblast differentiation and its downregulation in OS suggest a tumor suppressor activity of this gene in OS tumorigenesis. To test this hypothesis, we generated *FOXO1ER*-overexpressing stable cells and matching control cells. In this construct, the *FOXO1* coding sequence is fused in frame with the mutant ligand-binding domain of the estrogen receptor. Therefore, in the presence of 4-hydroxytamoxifen (4-OHT), FOXO1ER would translocate to the nucleus and regulate target gene transcription. We first verified the expression of FOXO1ER protein by immunoblotting (Figure 3a). The two cell lines expressed endogenous FOXO1 at a low level compared to the strong expression of the fusion protein. We then validated the nucleus translocation upon 4-OHT treatment (Figure 3b). In *FOXO1ER*-overexpressing stable cells, the protein was exclusively located in the cytoplasm. After addition of 4-OHT, the mutant FOXO1 protein was most seen in the nucleus. When FOXO1ER was activated by 4-OHT, we observed a significant reduction of viable cells in both OS cell lines (Figure 3c). Furthermore, colony formation assay in monolayer culture showed that activation of FOXO1 remarkably reduced the efficiency of colony formation (Figure 3d).

### FOXO1-induced cell cycle arrest and apoptosis

**Figure 4:**
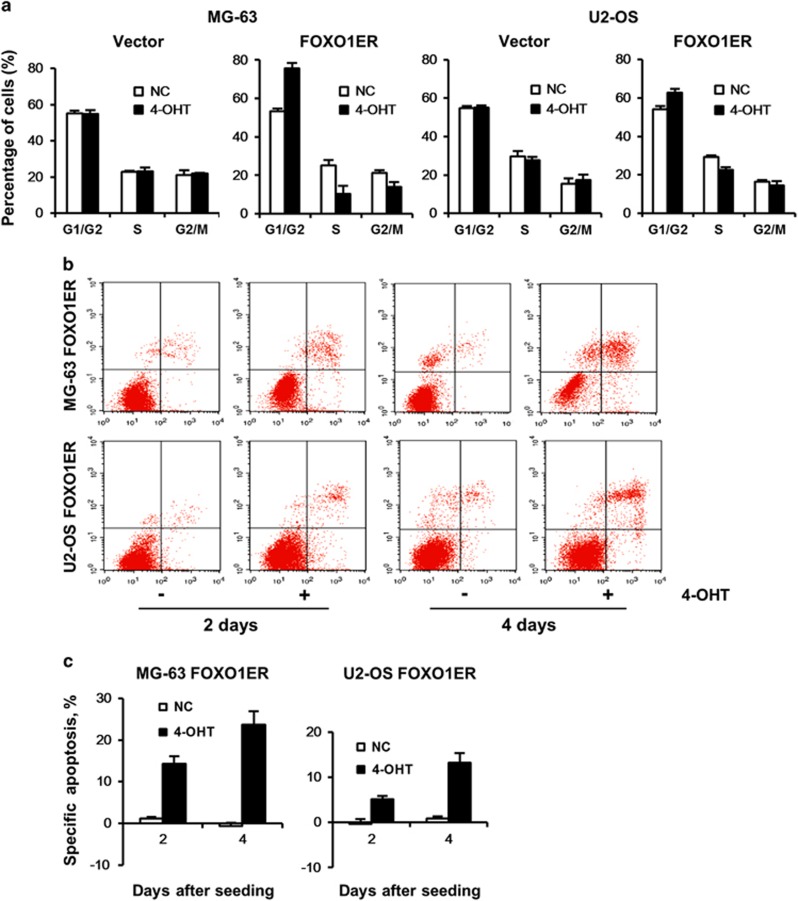
FOXO1 induces growth arrest and apoptosis in osteosarcoma cell lines. OS cell lines stably expressing the empty vector or *FOXO1ER* were seeded in six-well plates at a density of 2.5 × 10^5^ cells per well and treated with 4-OHT (100 nM) or vehicle. At different time points as indicated, cells were collected for cell cycle analysis (**a**) and apoptosis assay (**b**, **c**). (**a**) FOXO1 activation inhibits cell-cycle transition. After 24 h, cells were harvested and cell-cycle distribution was analyzed by propidium iodide staining. Bars represent the mean of three measurements±s.d. (**b**) FOXO1 induces cell apoptosis. Apoptosis was measured by annexin V-PE/7-AAD staining. The data are representative of at least three independent experiments that gave similar results. (**c**) The results of (**b**) are represented as specific apoptosis (SA): SA (%)=100(AE−AC)/(1−AC), where AE equals percentage of apoptotic cells in the experimental (4-OHT) group and AC equals percentage of apoptotic cells in the control (vehicle) group. Data are mean±s.d. of at least independent experiments.

The decrease in the viable cell numbers might be due to induction of cell cycle arrest and apoptosis by FOXO1.^31^ In both cell lines, we found that FOXO1 activation resulted in changes in cell-cycle parameters, with reduced percentage of cells in the S-phase accompanied by an accumulation of cells in G0/G1 phase (Figure 4a). Combining annexin V and 7-AAD staining (Figures 4b and c) and Hoechst 33258 staining (Supplementary Figure 3), we found that the decrease in the viable cell numbers could be partially attributed to the induction of apoptosis. Thus, FOXO1 affects growth of OS cell lines by induction of cell-cycle arrest and apoptosis.

### Inhibition of FOXO1 promoted OS cell survival and inhibited differentiation

**Figure 5:**
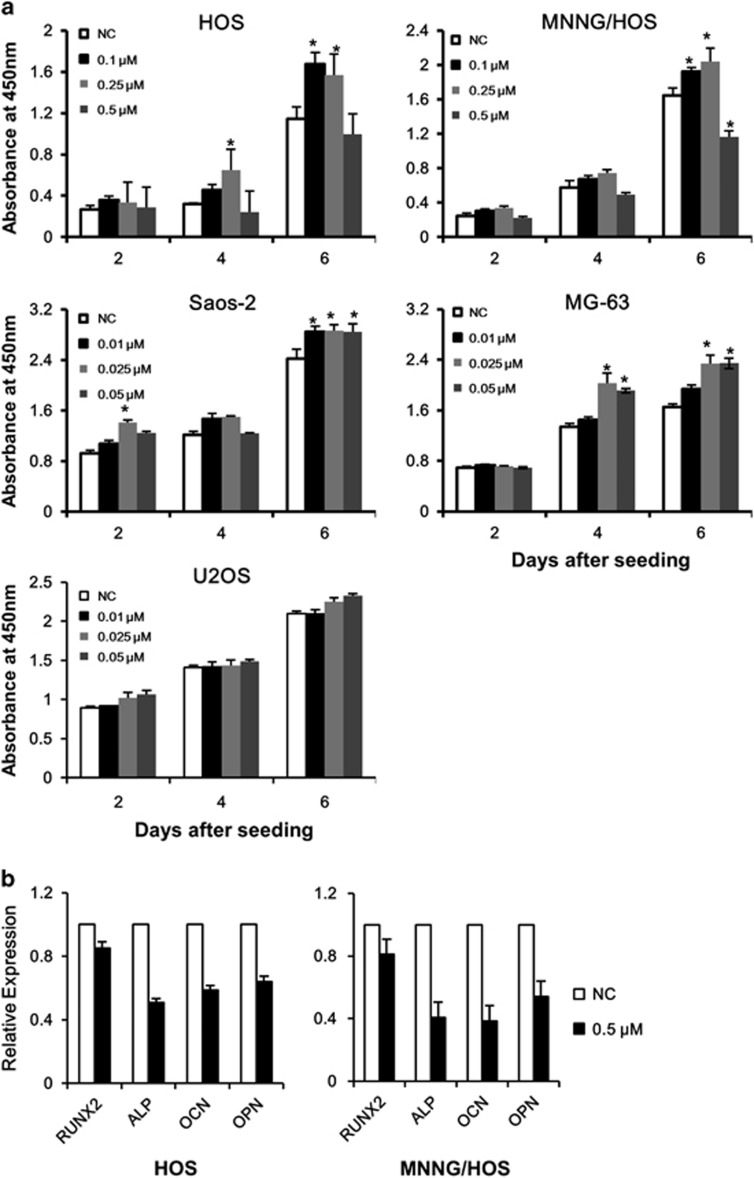
FOXO1 inhibition promotes cell growth and suppresses osteogenic differentiation. (**a**) OS cell lines were seeded in 96-well plates at a density of 1000 cells per well and treated with FOXO1 inhibitor as1842856 at the indicated concentrations. Cell viability was measured by CCK8. Data are mean±s.d. of at least three independent experiments. *P<0.05. (**b**) HOS and MNNG/HOS cell lines were seeded in six-well plates at a density of 2.5 × 10^5^ cells per well and treated with 0.1 μM as1842856 or vehicle. Osteogenic differentiation was induced by 50 μg/ml ascorbic acid and 10 mM β-glycerophosphate for 10 days. The gene expression of RUNX2, ALP, OCN and OPN was detected with quantitative RT-PCR. All experiments were repeated at least three times. The data represent mean±s.d. of at least three independent experiments.

To exclude the possibility that the impaired OS cell survival was due to supra-physiological overexpression of *FOXO1ER* transgene, we evaluated effects of FOXO1 inhibition on OS cell growth. We used AS1842856, a specific inhibitor of FOXO1 transcriptional activity.^32^ In the five OS cell lines tested, four cell lines demonstrated increased cell proliferation upon AS1842856 treatment (Figure 5). More importantly, the dose-responsive kinetics seemed to be correlate with FOXO1 expression: a) in cell lines with very low expression of FOXO1, AS1842856 at low concentrations induced moderate acceleration of cell growth (MG-63 and Saos-2) or no significant growth promotion (U2OS); b) in cell lines with moderate protein expression of FOXO1, AS1842856 at higher concentrations demonstrated more pronounced growth stimulation (HOS) (Figure 5a).

We next tried to evaluate the influence of FOXO1 activity on osteoblast differentiation of OS cells. During induction of osteoblast differentiation of both HOS and MNNG/HOS lines, we found that FOXO1 inhibition impaired expression of osteoblast marker genes including OCN (Osteocalcin/BGLAP), OPN (Osteopontin/SPP1) and ALP; the change of RUNX2 was not pronounced (Figure 5b), implicating that FOXO1 repression might be partially responsible for the blocked terminal differentiation observed in OS.

**Figure 6:**
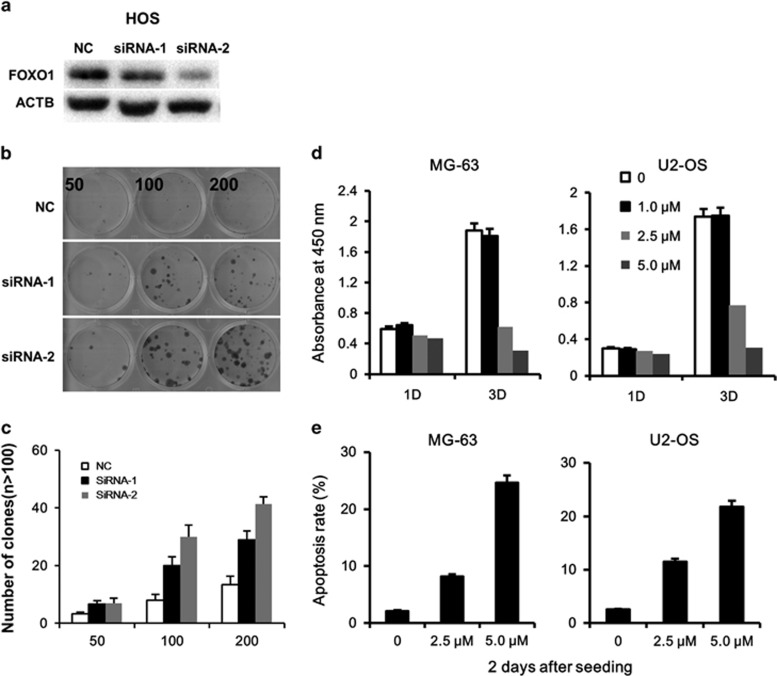
Modulation of FOXO1 activity influences OS cell survival. (**a**–**c**) FOXO1 knockdown promotes the capacity of colony formation. (**a**) FOXO1 expression after knockdown with two different siRNAs compared with the negative control (non-targeted siRNA). (**b**) Representative photograph of colony formation of HOS cells 12 days after plating. Cells were treated as described in Materials and Methods. (**c**) Quantitation of colony formation. All experiments were repeated at least three times. The data represent mean±s.d. of at least three experiments. (**d**, **e**) FOXO1 activation by MSA suppressed cell growth and promoted apoptosis in OS cells. (**d**) OS cells were seeded in 96-well plates at a density of 1000 cells per well and treated with MSA at the indicated concentrations. Cell viability was measured by CCK8. The data represent mean±s.d. of at least three experiments. (**e**) MG-63 and U2-OS cells were seeded in six-well plates at a density of 2.5 × 10^5^ cells per well and treated with MSA at the indicated concentrations. Cell apoptosis was measured by annexin V-PE/7-AAD staining 2 days later. The data represent mean±s.d. of at least three experiments.

We also evaluated the consequence after knockdown of FOXO1 expression by siRNA. Two siRNAs were designed and used, both achieved pronounced repression of FOXO1 expression, with siRNA-2 being the more efficient one (Figure 6a). Repressed FOXO1 protein expression resulted in increased colony formation capability. Cells transfected with siRNA-2 formed more colonies, which was correlated with the higher efficiency of knockdown (Figures 6b and c).

### Activation of FOXO1 by a chemical compound influenced OS cell survival

Methylseleninic acid (MSA) is a small-molecule activator of FOXO1 that had been shown to activate FOXO1 in prostate cancer cells and to increase FOXO1 expression in Ewing sarcoma cells paralleled by a significant decrease in ES tumor growth.^33, 34^ To test if FOXO1 could be a valid therapeutic target in OS, we explored the effects of MSA on OS cells. MG-63 and U2-OS cells were treated with MSA or vehicle. Cell proliferation was detected by a CCK8 colorimeter. MSA significantly suppressed cell growth at concentrations of 2.5 and 5.0 μM (Figure 6d). Combining annexin V and 7-AAD staining, we found that MSA induced apoptosis in both OS cell lines (Figure 6e).

### Modulation of target gene expression and Wnt/β-catenin signaling by FOXO1

**Figure 7:**
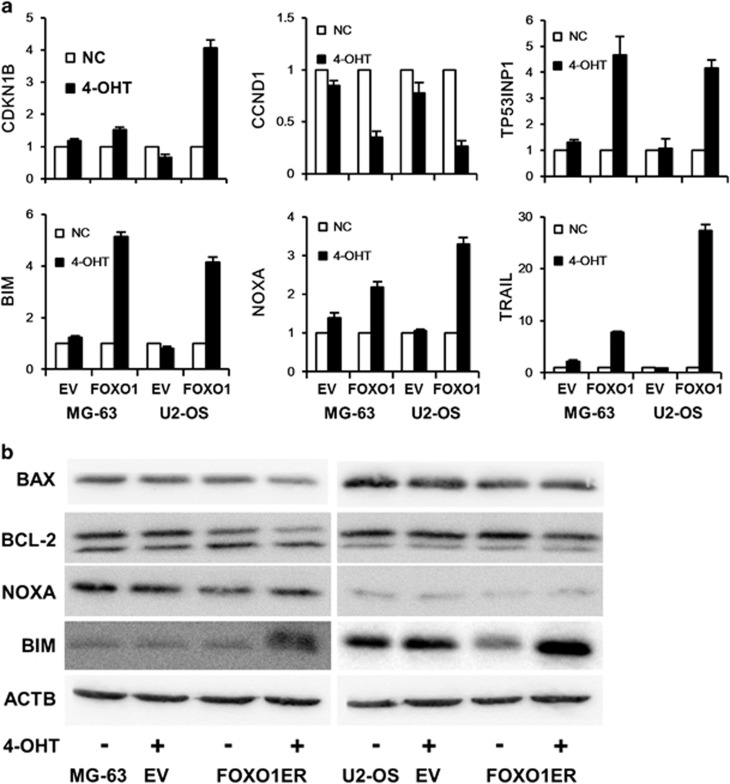
FOXO1 regulates target gene expression. MG-63 and U2-OS cells expressing *FOXO1ER* or empty vector were treated with 100 nM 4-OHT or vehicle. One day later, cells were harvested, mRNA and protein was prepared. (**a**) CDKN1B, CyclinD1, TP53INP1, BIM, NOXA and TRAIL mRNA expression was assessed. EV indicates empty vector. The data represent mean±s.d. of least three experiments. (**b**) The expression of BAX, BCL-2, NOXA and BIM protein was detected by immunoblotting. Anti-ACTB antibody was used as a loading control.

In various model systems, FOXO1 acts as a tumor suppressor by multiple mechanisms including regulating expression of target genes involved in control of cell-cycle progression and apoptosis. We measured expression of target genes of FOXO1. FOXO1 activation increased expression of *CDKN1B/p27*, *TP53INP1*, *BIM*, *NOXA* and *TRAIL*, and decreased CCND1/Cyclin D1 expression (Figure 7a). At the protein level, BIM expression was significantly upregulated (Figure 7b).

**Figure 8:**
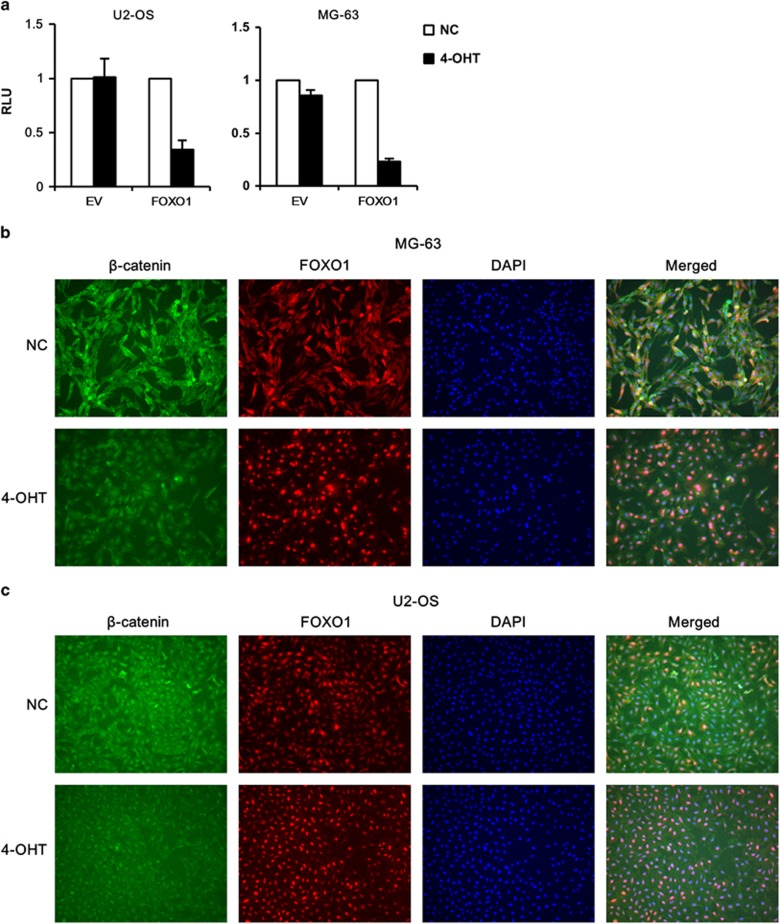
FOXO1 represses Wnt/β-catenin signaling. MG-63 and U2-OS cells expressing FOXO1ER or empty vector were treated with 100 nM 4-OHT or vehicle. (**a**) One day later, cells were co-transfected with TOPflash luciferase construct and Renilla luciferase vector; the luciferase activity was measured 24 h after transfecting. All experiments were carried out in triplicate. The data represent mean±s.d. of least three experiments. (**b**, **c**) Two hours (data not shown) or 24 h later, cells were fixed and immunostained with FOXO1 (red) and β-catenin (green) antibodies and visualized under a microscope. 4-OHT induced FOXO1 translocation from the cytoplasm to the nucleus in MG-63 FOXO1ER cells (**b**) and U2-OS FOXOER cells (**c**), and decreased the expression of β-catenin with no influence on the intracellular localization of β-catenin. The experiment was repeated three times with similar observations.

As FOXOs were shown to antagonize canonical Wnt/β-catenin signaling, leading to impaired expression of target genes including CCND1 and repressed proliferation of committed osteoblast precursors,^11^ we measured Wnt/β-catenin activity in FOXO1ER lines. FOXO1 activation significantly impaired Wnt/β-catenin activity in both cell lines as judged from the TOPflash/Renilla intensity (Figure 8a). We then asked whether FOXO1 caused nuclear exclusion and degradation of β-catenin. We investigated the effects of FOXO1 on the cellular localization of β-catenin. OS cells stably expressing FOXO1ER were treated with 4-OHT (100 nM) for 2  or 24 h. Cells were then fixed and immunostained with FOXO1 (red) and β-catenin (green) antibodies. 4-OHT induced FOXO1ER translocation from the cytoplasm to the nucleus. However, nuclear translocation of FOXO1ER did not influence the cellular localization of β-catenin. Interestingly, we found that FOXO1ER activation inhibited the expression of β-catenin as the fluorescence intensity decreased significantly 24 h after 4-OHT treatment (Figures 8b and c).

## Discussion

In the present study, we found that FOXO1 was generally low or absent in OS, with a minority of cases having moderate expression. The *FOXO1* locus was frequently involved in copy number variation and loss of heterozygosity in OS, indicating chromosomal aberrations might be partially responsible for the low *FOXO1* expression in some OS cases. FOXO1 activation in OS cell lines led to cell cycle arrest and apoptosis that was associated with modulation of target genes including BIM and repressed Wnt/β-catenin signaling. FOXO1 inhibition promoted cell proliferation and attenuated osteogenic differentiation of OS cells. Restoration of FOXO1 activity by a chemical compound MSA led to impaired proliferation and apoptosis.

The human *FOXO1* and *RB1* genes are located in 13q14, a locus with recurrent losses in OS.^27, 28, 29^ More importantly, patients with losses of 13q14 had significantly lower event-free survival.^28^ In the present study, analysis of whole-genome sequencing revealed 5 gains, 6 losses and 15 cases of loss of heterozygosity of the *FOXO1* locus in 34 OS cases, all of which are broad-scale changes (over 10 Mb). Therefore, the genomic alteration might also involve *RB1* (the distance between the two gene loci is about 8 Mb). A monoallelic loss of *RB1* and *FOXO1* was identified in some cases of cellular angiofibromas, spindle cell lipomas and myofibroblastomas, all of which are benign stromal tumors.^35, 36^ Whether OS is another stromal tumor with some cases having loss of *RB1* and *FOXO1* deserves further study. Gains of 13q14 have never been reported in OS; further efforts might be needed to ascertain the significance of this finding. In the present study, no point mutations in the coding sequence or DNA hypermethylation in the promoter region of *FOXO1* were identified, which is in line with previous studies showing *FOXO1* might be involved in genomic loss, but rarely involved in mutation or DNA hypermethylation in tumorigenesis.^31, 37, 38^

FOXOs have complex roles in development and tumorigenesis. In the hematopoietic lineage, FOXOs enhance survival of hematopoietic stem cells, inhibit myeloid lineage expansion and promote lymphoid development.^39^ In addition, FOXOs promote leukemia-initiating cell function *in vivo* and improve the survival of animals with acute myeloid leukemia;^40^ we and others found that FOXOs act as tumor suppressors in lymphomagenesis.^31, 38, 41^ During bone development, in the early progenitors of the osteoblast lineage targeted by the *Mx1-Cre* transgene, FOXOs promote maintenance and differentiation by activation of Runx2;^20^ FOXOs repress proliferation of Osx1^+^ committed osteoblast precursors by inhibiting the canonical Wnt/β-catenin signaling.^11^ FOXO1 is vital for osteocyte survival through reduction of ROS generation.^17^ Therefore, theoretically, FOXOs might play a tumor-suppressing or oncogenic role in OS pathogenesis. In the present study, modulation of FOXO1 activity did not lead to marked change of Runx2 expression (Figure 5b and data not shown). We found that FOXO1 repressed survival of OS cells partially by inhibition of the Wnt/β-catenin signaling, demonstrating that the inhibition of Wnt/β-catenin signaling by FOXOs is preserved in bone development and tumorigenesis. We found that FOXO1 had no significant influence on the subcellular localization of β-catenin; instead, FOXO1 inhibited expression of β-catenin. Further efforts are necessary to elucidate the mechanisms responsible for FOXO1-induced downregulation of β-catenin.

Loss of differentiation is a hallmark of cancer including OS. Especially, well-differentiated OS is classified as low-grade and has better prognosis, while dedifferentiated OS usually falls into the high-grade category with an inferior prognosis. We found that FOXO1 activation induced cell cycle arrest and its inhibition impaired osteogenic differentiation of OS cell lines, which is in line with a recent study showing FOXO1 is a positive promoter of osteoblastogenesis *in vitro*.^42^ Our data implicate that FOXO1 deficiency might contribute to the disrupted terminal differentiation observed in the OS tumors.

In conclusion, our data provide evidence supporting the tumor-suppressing roles played by FOXO1 in OS tumorigenesis. We believe restoration of FOXO1 activity could be a potential therapeutic strategy for OS therapy.

## Materials and methods

### Cell culture and treatments

OS cell lines MG-63, U2-OS, Saos-2 and a chondrosarcoma cell line SW1353 were all purchased from the cell bank of the Chinese Academy of Sciences and cultured in DMEM/F12. OS cell lines HOS and MNNG/HOS were cultured in α-MEM. The Platinum-A retroviral packaging cell line was purchased from Cell Biolabs, Inc. (San Diego, CA, USA) and grown in DMEM/high glucose following the protocol from the manufacturer. The medium was supplemented with 10% fetal bovine serum, 100 μg/ml streptomycin and 100 U/ml penicillin. Cultures were maintained at 37 °C in a humidified incubator in an atmosphere of 5% CO_2_. The FOXO1 inhibitor AS1842856 was described earlier^32^ and purchased from Calbiochem (Merck Millipore, Billerica, MA, USA). Osteogenic differentiation of HOS and MNNG/HOS cells was induced by 50 μg/ml ascorbic acid and 10 mM β-glycerophosphate. Ascorbic acid and β-glycerophosphate were purchased from Sigma-Aldrich (St Louis, MO, USA).

### Isolation and culture of hMSCs

Human bone marrow were obtained from three health volunteers after the donors had given their written informed consent. All procedures were approved by the ethics committee of our institute. Briefly, bone marrow aspirates were passed through a 70-μm nylon mesh, and diluted in culture medium. The mononuclear cells were isolated by Ficoll density gradient centrifugation (1.077 g/ml, Sigma, Taufkirchen, Germany) at 400 *g* for 35 min. Mononuclear bone marrow cells were plated in culture flasks, and hMSCs were isolated by their adherence to plastic. Passage 2–4 cells were used for experiments. hMSCs were validated for their capabilities to differentiate into osteoblasts, adipocytes and chondrocytes. Osteogenic differentiation was induced by OriCell Human Mesenchymal Stem Cell Osteogenic Differentiation Medium (Cyagen Biosciences Inc., Santa Clara, CA, USA).

### Retroviral vectors and infection of OS cell lines

The pCFG5-IEGZ retroviral vectors have been described earlier.^43^ The construct pCFG5-FOXO1(A3)ER contains a FOXO1-ER fusion gene, a constitutively active form of human FOXO1 fused in-frame with a modified estrogen receptor (ER) ligand-binding domain, as described earlier.^31, 44^ The FOXO1-ER fusion protein can be specifically activated by 4-OHT. Platinum-A, the retroviral packaging cell line, was transfected with retroviral vectors using Lipofectamine (Life Technologies). Forty-eight and 72 h after transfection, GFP expression was evaluated under fluorescent microscopy to determine the transfection efficiency. Supernatants were collected and supplemented with 8 μg/ml polybrene, and used for infection of MG-63 and U2-OS cells. Infected cells were selected with 50 μg/ml Zeocin (Calbiochem) until the percentage of GFP-expressing cells determined by flow cytometric analysis was above 95%. FOXO1-ER protein was activated by 4-OHT (Calbiochem).

### RNA interference

Three small interfering RNAs (siRNAs) specific for human FOXO1 were designed and synthesized by Ribbio Co. Ltd (Q000002308-1-A, Guangzhou, China). Two of them were selected for their efficiency of knockdown. Scrambled non-targeting siRNA was used as a negative control. HOS cells were transfected with X-tremeGENE siRNA Transfection Reagent (Roche, Mannheim, Germany) according to the manufacturer's instructions. After transfection, cells were cultured for 12 h and then seeded in 12-well plates at a density of 50, 100, 200 cells per well. The transfection was performed every 4 days. Twelve days later, the plates were stained with 0.1% crystal violet (Biosharp, Hefei, China) and the colony numbers were counted under a microscope.

### Immunofluorescence analysis

OS cells expressing *FOXO1ER* transgene were processed for immunofluorescence analysis. Briefly, cells were seeded in 96-well plates at a density of 3.0 × 10^3^ cells per well and treated with 4-OHT (100 nM) or vehicle for 2 or 24 h, then fixed with 4% paraformaldehyde solution for 20 min at room temperature and permeabilized with 0.2% Triton X-100 solution for 5 min. Subsequently, cells were blocked with 5% BSA for 1 h, incubated with monoclonal rabbit anti-FOXO1 (1:200 dilution, Cell Signaling Technology, #2880) and monoclonal mouse anti-β-Catenin (1:200 dilution, Cell Signaling Technology, #2677) at 4 °C overnight. Next, detection of the bound primary antibodies was enabled by incubating cells with secondary red-fluorescent Alexa Fluor 594 goat anti-rabbit IgG antibody (A11012, Molecular Probes, Eugene, OR, USA) and green-fluorescent Alexa Fluor 488 goat anti-mouse IgG antibody (A11001, Molecular Probes, Eugene, OR, USA) for 1 h at room temperature. Finally, Microscopic observation was performed using a laser scanning confocal microscopy (FV500, OLYMPUS Co., Japan).

### Cell proliferation assay and colony formation assay

Cell proliferation assays were done as described earlier.^45^ For colony formation assay, cells were plated in 12-well plates at 50, 100, 200 cells per well, 4-OHT or vehicle was added every 48 h. After 2 weeks, the plates were fixed in 4% paraformaldehyde and stained with 0.1% crystal violet (Biosharp, Hefei, China). The number of colonies was counted manually.

### Apoptosis and cell cycle analysis

For apoptosis analysis, 1 × 10^5^ cells were co-stained with annexin V-PE and 7-amino-actinomycin D (7-AAD, both from Biolegend, San Diego, CA, USA). Cell death was measured by a Flow Cytometer FACSCalibur (BD Biosciences, San Jose, CA, USA) as described before.^46^ To determine cell-cycle distribution, 1 × 10^6^ cells were fixed and stained with 70% cold ethanol and propidium iodide (PI). DNA contents were measured by flow cytometry. Data were analyzed using ModFit cell-cycle analysis software (Verity Software House).

### Quantitative RT-PCR

Quantitative real-time polymerase chain reaction (qRT-PCR) was performed as described before.^47^ Briefly, total RNA was isolated from OS cell lines and hMSC using TRIzol reagent (Invitrogen, Carlsbad, CA, USA) and first-strand cDNA was synthesized with MMLV reverse transcriptase (Promega, Madison, WI, USA). Templates were amplified using QuantiTect SYBR Green PCR Kit (Qiagen, Hilden, Germany) on the iCycler real time PCR instrument (Bio-Rad, Hercules, CA, USA). The primers were synthesized by Invitrogen; sequences 5′ to 3′, sense and antisense, were as follows: FOXO1: GGGCCCTAATTCGGTCATGT and CGCCCGTTAACTGCAGATGT; TRAIL: TTCCTGAGCAACTTGCACTT and TGGACCATTTGTTTGTCGTT-3′ BIM: ACTCTCGGACTGAGAAACGC and CCTTCTCGGTCACACTCAGA; CCND1: CATTGATTCAGCCTGTTTGG and GAATTCATCGGAACCGAACT; CDKN1B: TCAAACGTAAACAGCTCGAATTA and ACAGGATGTCCATTCCATGA; TP53INP1:TTCCTCCAACCAAGAACCAGA and GCTCAGTAGGTGACTCTTCACT; OCN (Osteocalcin/BGLAP): CACTCCTCGCCCTATTGGC and CCCTCCTGCTTGGACACAAAG; OPN (Osteopontin/SPP1): CTCCATTGACTCGAACGACTC and CAGGTCTGCGAAACTTCTTAGAT; PMAIP1 (NOXA): GCTGGAAGTCGAGTGTGCTA and CAGTCAGGTTCCTGAGCAGA; RUNX2: TGGTTACTGTCATGGCGGGTA and TCT-CAGATCGTTGAACCTTGCTA; ALP: ACCACCACGAGAGTGAACCA and CGTTGTCTGAGTACCAGTCCC; GAPDH: TTCCAATATGATTCCACCCA and GATCTCGCTCCTGGAAGATG.

### Immunoblot

Immunoblot was done as described earlier.^45^ The following first antibodies were from Cell Signaling Technology (Boston, MA, USA): FOXO1 (#2880), BCL-2 (#2870), BAX (#5023), BIM (#2933), NOXA (#14766), TRAIL (#3219), and β-catenin (#2677). The antibody against β-actin was from Sigma-Aldrich (A5060). As second antibody we used goat anti-rabbit IgG-HRP (sc-2004, Santa Cruz Biotechnology, Santa Cruz, CA, USA). Signals were visualized with enhanced chemiluminescence according to the manufacturer's recommendations.

### Human material and immunohistochemistry

Sixty-two OS specimens and 18 osteoid osteoma specimens were included in this study. Approval for these studies was obtained from the ethics board of our institute. All cases had detailed clinical data and none received preoperative chemotherapy or radiotherapy. All samples were drawn from our archive of formalin-fixed, paraffin-embedded tissues. For immunostaining, the slides were deparaffinized, rehydrated, then immersed in 3% hydrogen peroxide solution for 20 min, and then pretreated in a micro-wave oven in 0.125% trypsin for 2 min. The slides were blocked by 5% BSA at 37 °C for 30 min, and then incubated with rabbit monoclonal antibody against FOXO1 (1:50 dilution, Cell Signaling Technology, #2880) overnight at 4 °C. After washing with PBS three times, the slides were incubated with horseradish peroxidase-conjugated secondary antibody (diluted 1:100) for 1 h at room temperature. Peroxidase activity was visualized by a mixture of DAB solution for 10 min, rinsed in deionized water and then counterstained with hematoxylin.

### Luciferase reporter assay

MG63-FOXO1ER and U2OS-FOXO1ER cells were maintained in DMEM/F12 and cells of 70% confluence in 96-well plates were treated with 4-OHT at a concentration of 100 nM for 24 h, and then were transfected with 100 ng of the TOPflash luciferase construct and 10 ng of the Renilla luciferase vector with X-tremeGENE HP DNA Transfection Reagent (Roche). Cell extracts were prepared 24 h after transfection, and the luciferase activity was measured with the Dual-Luciferase Reporter Assay system (Promega). Values of TOPflash luciferase activity were normalized to Renilla activity.

### Statistical analysis

All experiments were independently repeated three times. Data were expressed as mean±s.d. One-way analysis of variance (ANOVA) with subsequent Student–Newman–Keuls test was used to determine significant differences in multiple comparisons. All statistical analyses were carried out with SPSS13.0 software (SPSS, Chicago, IL, USA), *P*<0.05 was considered statistically significant.

## Figures and Tables

**Table 1:** Expression of FOXO1 in benign and malignant bone tumors

*Neoplasm type*	−	+	++	+++	*Total cases*
Osteoid osteoma	5	10	3	0	18
Osteosarcoma	39	15	8	0	62

−, negative staining; +, weak staining; ++, moderate staining; and +++, strong staining.

**Table 2:** Expression of FOXO1 in chondromatous, fibromatous and osteogenous areas of osteosarcoma

*Areas*	−	+	++	*Total cases*
Chondromatous	8	3	3	14
Fibromatous	12	7	3	22
Osteogenous	8	1	0	9

−, negative staining; +, weak staining; ++, moderate staining; and +++, strong staining.
