# Supplementary material for: FOXO1 inhibits osteosarcoma oncogenesis via Wnt/β-catenin pathway suppression
Source: Oncogenesis. 2015 Sep 7;4(9):e166–. doi: 10.1038/oncsis.2015.25 (PMC4767937; doi:10.1038/oncsis.2015.25)
Supplement: Supplementary Information [file oncsis201525x1.doc]

FOXO1 inhibits osteosarcoma oncogenesis via Wnt/β-catenin pathway suppression.

Hanfeng Guan^1^,^a^, Peng Tan^1,a^, Linka Xie^2^, Baoguo Mi^1^, Zhong Fang^1^, Jing Li^1^, Junqiu Yue^3^, Hui Liao^1^, Feng Li^1*^

**Supplementary information.**

**Supplemental Figure 1**

**
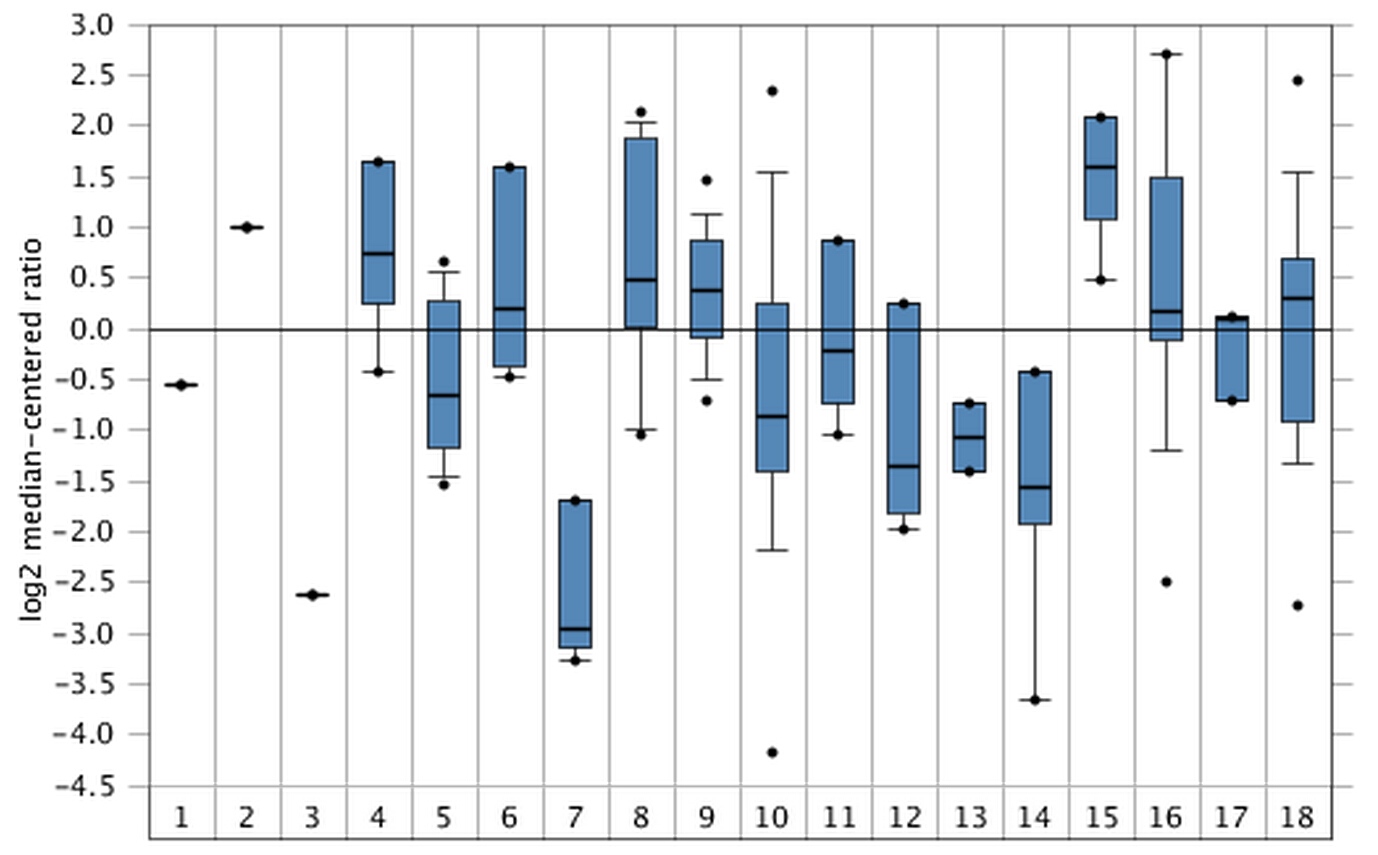
**

**Supplemental Figure 1. Expression of *FOXO1* in sarcomas.** With help of a oncomine software (https://www.oncomine.org), we analyzed published gene expression data of 181 tumors including 16 types of human bone and soft tissue sarcomas (http://www.ncbi.nlm.nih.gov/geo/query/acc.cgi?acc=GSE2553) . We found that expression of FOXO1 was very low in gastrointestinal stromal tumor and OS comparing with other sarcomas (3 types of sarcomas with only 1 case were not analyzed). 1: Alveolar Soft Part Sarcoma (1 case), 2: Chondrosarcoma (1 case), 3: Clear Cell Sarcoma of Soft Tissue (1 case), 4: Dermatofibrosarcoma Protuberans (5 cases), 5: Ewing's Sarcoma (19 cases), 6: Fibrosarcoma (7 cases), 7: Gastrointestinal Stromal Tumor (5 cases), 8: Leiomyosarcoma (17 cases), 9: Liposarcoma (33 cases), 10: Malignant Fibrous Histiocytoma (38 cases), 11: Malignant Hemangiopericytoma (6 cases), 12: Malignant Peripheral Nerve Sheath Tumor (6 cases), 13: Mixed Mesodermal (Mullerian) Tumor (2 cases), **14: Osteosarcoma (5 cases)**, 15: Rhabdomyosarcoma (6 cases), 16: Previously unclassified sarcomas (10 cases), 17: Schwannoma (3 cases), 18: Synovial Sarcoma (16 cases).

**Supplemental Figure 2**

**
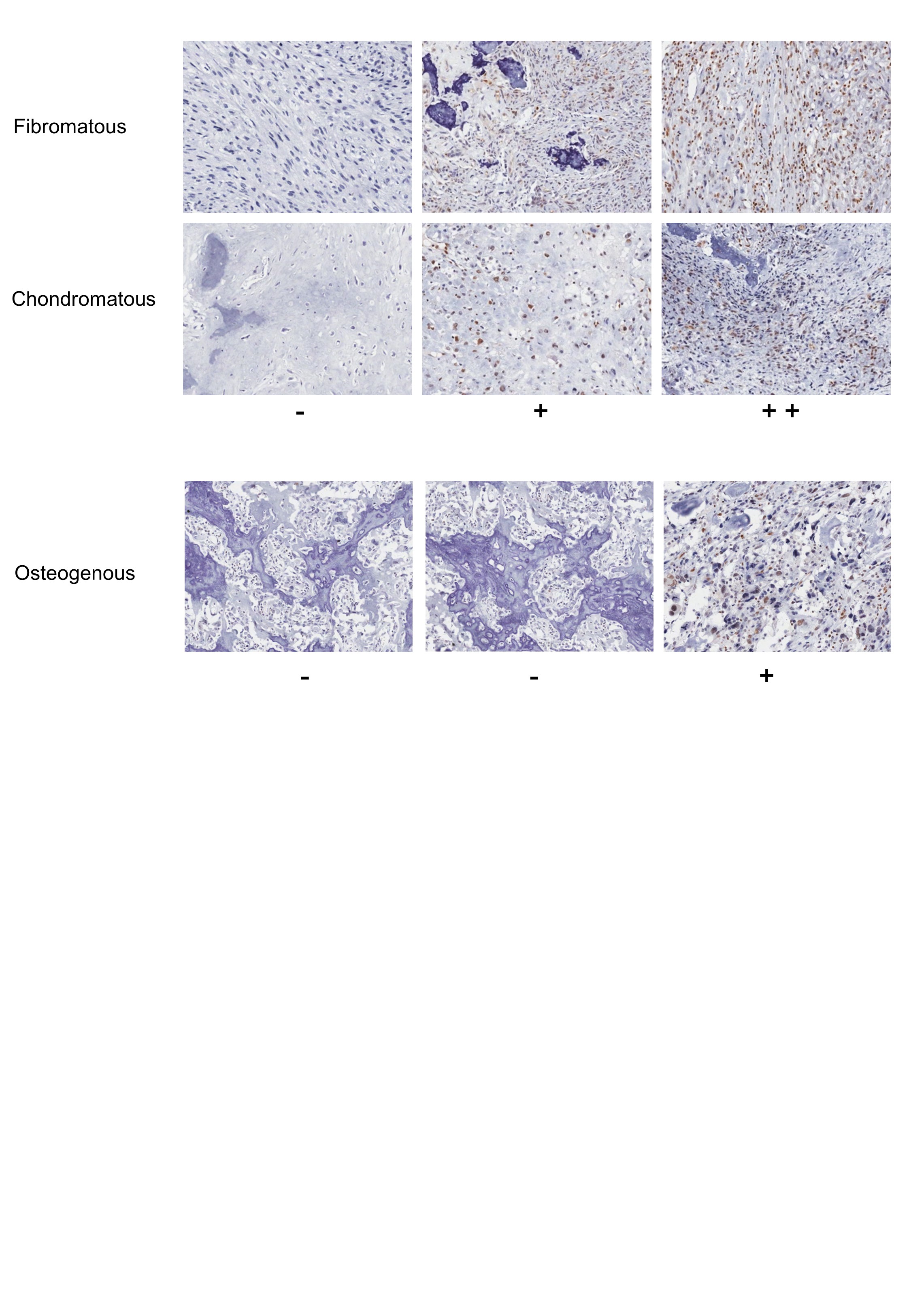
**

**Supplemental Figure 2. Expression of FOXO1 in chondromatous, fibromatous, osteogenous areas of osteosarcoma.**

**Supplemental Figure 3**


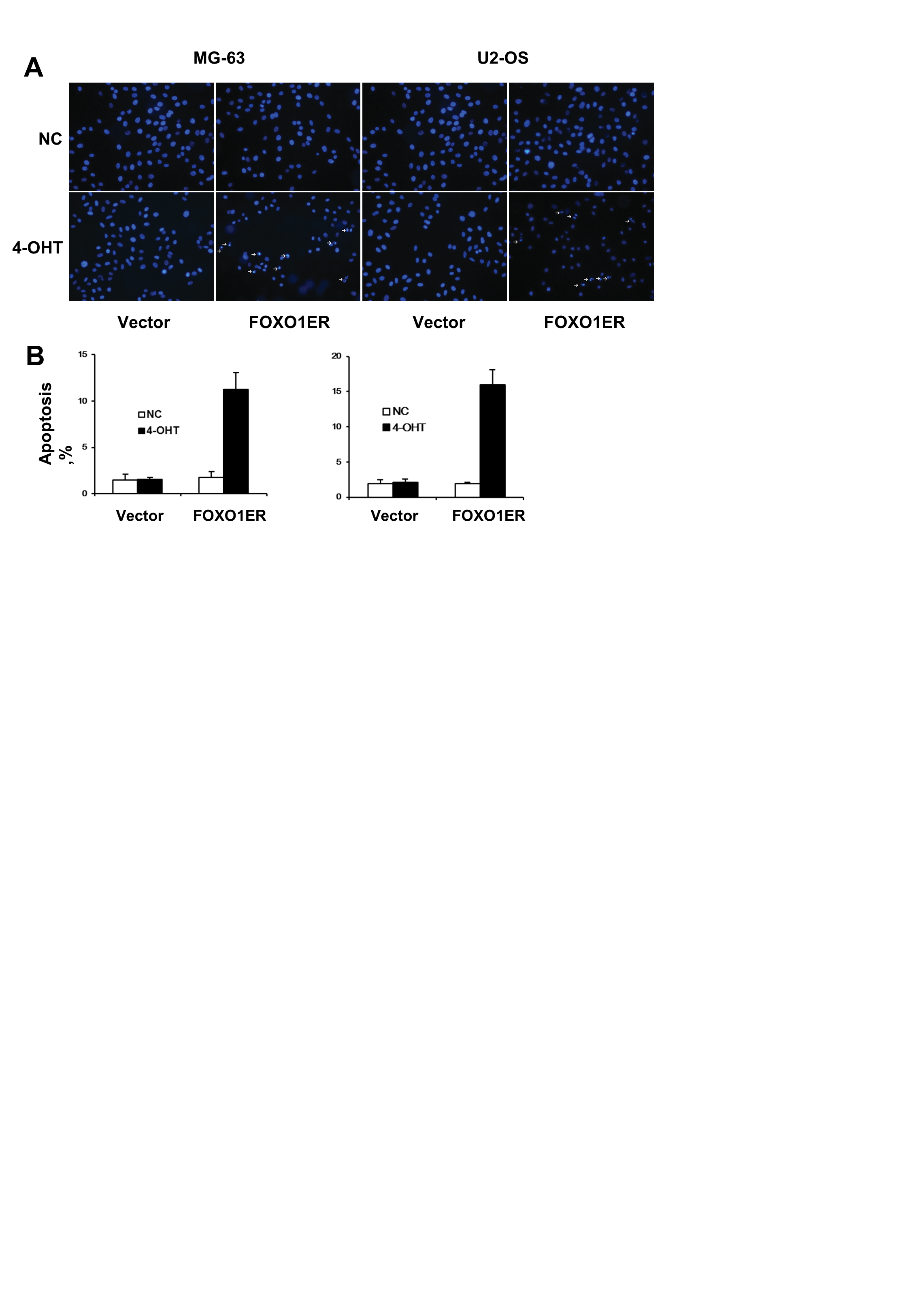


**Supplemental Figure 3. FOXO1 induces apoptosis in OS cells.** For Hoechst 33258 staining, MG-63 and U2OS cells expressing FOXO1ER or empty vector were seeded on coverslips on a 12-well plate at a density of 0.5×10^5^ per well. After 24 h incubation with vehicle or 4-OHT (100 nM), cells were fixed in 4% [paraformaldehyde](app:ds:paraformaldehyde) and then stained with Hoechst 33258 dye (Beyotime Institute of Biotechnology,Jiangsu, China) for 10 minutes. Thereafter, cells were washed with PBS, mounted on slides and observed under a fluorescence microscope. Positive staining cells that represented apoptotic cells with DNA condensation were indicated with the white arrows (A). (B) Five hundred cells were counted per treatment. All experiments were repeated at least 3 times. The data represent mean±SD of at least 3 experiments.

**REFERENCES**

1. Baird K, Davis S, Antonescu CR, Harper UL, Walker RL, Chen Y, et al. Gene expression profiling of human sarcomas: insights into sarcoma biology. Cancer Res. 2005;65(20):9226-35.
